# Potential therapeutic benefit of exogenous ketone ester administration in delirium: a narrative review

**DOI:** 10.1186/s13054-025-05680-5

**Published:** 2025-10-07

**Authors:** Ryan Smith, Fiona Harrison, Julie Bastarache, Shawniqua Williams Roberson, Elma Zaganjor, Pratik Pandharipande, Todd Rice, Wes Ely

**Affiliations:** 1 Critical Illness, Brain Dysfunction, and Survivorship Center, Center for Health Services Research, Vanderbilt University Medical Center, Nashville, TN USA; 2 Division of Allergy, Pulmonary, and Critical Care Medicine, Department of Medicine, Vanderbilt University Medical Center, Nashville, TN USA; 3 Division of Diabetes, Endocrinology & Metabolism, Department of Medicine, Vanderbilt University Medical Center, Nashville, TN USA; 4 Department of Neurology, Vanderbilt University Medical Center, Nashville, TN USA; 5 Department of Biomedical Engineering, Vanderbilt University, Nashville, TN USA; 6 Department of Molecular Physiology and Biophysics, Vanderbilt University School of Medicine, Nashville, TN USA; 7 Department of Anesthesiology, Division of Anesthesiology Critical Care Medicine, Vanderbilt University Medical Center, Nashville, TN USA; 8 Geriatric Research, Education and Clinical Center (GRECC) Service, Department of Veterans Affairs Medical Center, Tennessee Valley Healthcare System, Nashville, TN USA

**Keywords:** Delirium, Exogenous ketone ester, Beta-hydroxybutyrate, Neurometabolism, Neuroinflammation, Critical illness, Intensive care unit

## Abstract

Delirium is a prevalent neuropsychiatric syndrome during critical illness and is associated with prolonged hospitalization, increased mortality, and post-ICU cognitive decline. It is hypothesized to result from systemic inflammation, disrupted neurotransmission, and failure of cerebral energy metabolism. This narrative review highlights the key role of altered neurometabolism and neuroinflammation, which occurs due to peripheral inflammation, compromised blood-brain barrier integrity, and increased microglial glycolysis. These changes limit neuronal glucose uptake, leading to a brain energy crisis and consequently amplifying oxidative and inflammatory stress. We focus on studies of ICU delirium in the setting of acute critical illness with an emphasis on sepsis-associated encephalopathy, where mechanistic data derived from murine models are most robust. Ketones bypass the glycolytic bottleneck and enter the tricarboxylic acid cycle directly, activating signaling pathways that enhance mitochondrial biogenesis, bolster antioxidant defenses, modulate neurotransmission, and reduce inflammation. In models of neurodegenerative diseases and traumatic brain injury, ketosis restores cerebral metabolism, reduces neuroinflammation, and enhances cognitive function. Additionally, preliminary human studies have demonstrated cognitive benefits and patient tolerance of ketone supplementation. Although data in the critically ill are limited, pilot studies suggest that enteral ketone supplementation can safely achieve therapeutic serum concentrations without worsening acidosis or hemodynamic instability. We hypothesize that exogenous ketone ester supplementation may support brain energy production by providing an alternative substrate for energy production, reducing microglial substrate competition, and mitigating the neuronal stress that precipitates delirium. In conclusion, exogenous ketone esters are a biologically plausible, rapidly acting metabolic intervention that warrants rigorous clinical evaluation as a novel strategy to prevent or treat delirium in those who are critically ill. However, randomized controlled trials are essential for verifying safety, determining optimal dosing, and assessing clinical effectiveness in the intensive care setting.

**Graphical abstract:**

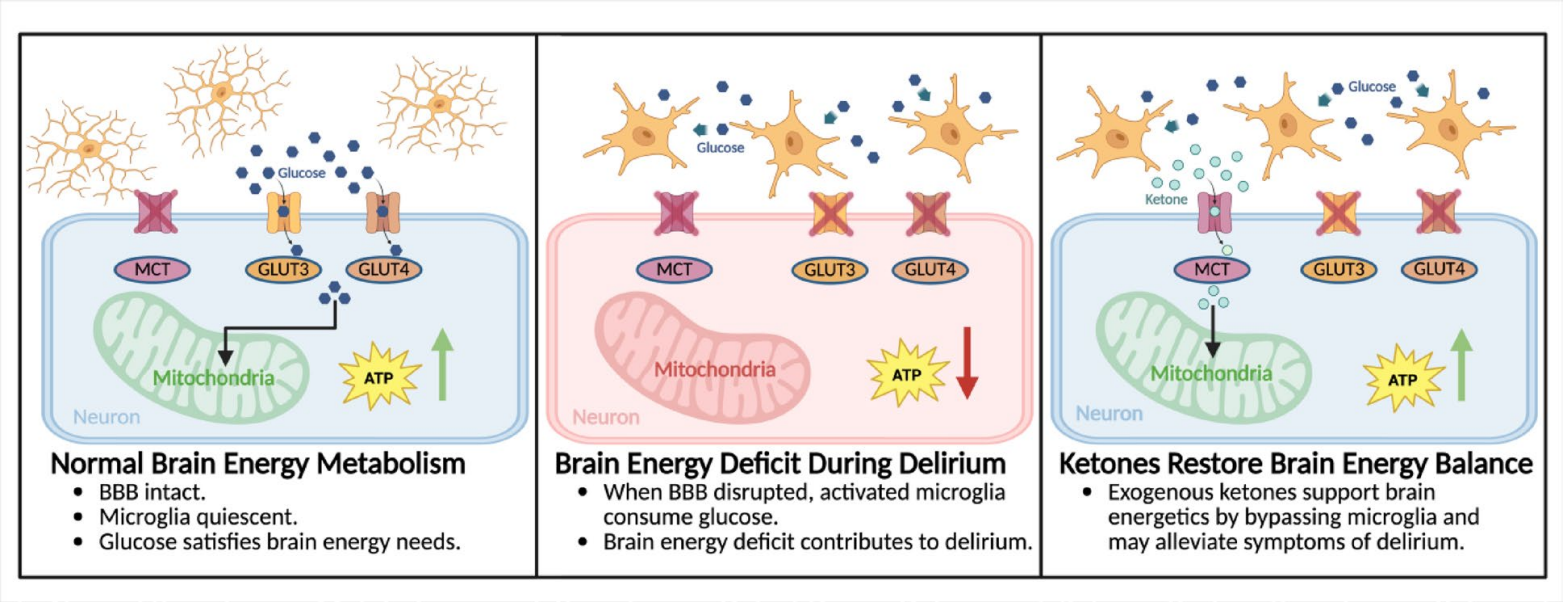

## Introduction

Delirium is a prevalent neuropsychiatric syndrome characterized by an acute disturbance in attention, cognition, and consciousness [1]. It is associated with significant morbidity, mortality, and healthcare expenditures. The pathophysiology of delirium is hypothesized to involve systemic inflammation, neurotransmitter imbalance, and altered neurometabolism, which refers to the biochemical processes the brain uses to produce and regulate energy to support neuron function. Despite advances in understanding the mechanisms underlying delirium, effective treatments remain elusive. This supports the need for novel therapeutic approaches. Ketones have shown early promise as treatments for traumatic brain injury and several neurodegenerative diseases. They are produced in the liver during periods of low glucose availability, such as fasting or carbohydrate restriction, and serve as an alternative energy source for the brain. Ketones support brain energetics by bypassing glucose metabolism, reducing oxidative stress, and decreasing inflammation. This review explores the potential therapeutic role of ketones as a treatment for delirium to spark a pathway of discovery to determine answers to existing questions.

## Neurometabolism in delirium

The pathophysiology of delirium is complex and incompletely understood; however, studies have long suggested that aberrant cerebral metabolism may play a role [1, 2]. Recent research has provided further evidence supporting the connection between brain metabolism and delirium. During states of increased systemic inflammation, such as sepsis or trauma, the brain experiences a mismatch between energy supply and demand, which is commonly associated with delirium in those with preexisting cognitive impairment [3]. In patients with hip fracture, elevated cerebrospinal fluid (CSF) levels of both lactate and ketones have been associated with delirium; these metabolic changes were independent of systemic and CSF glucose levels [3, 4]. Furthermore, FDG-PET imaging demonstrates a significant reduction in cerebral metabolism across cortical and subcortical regions in delirium, even when controlling for dementia and severity of acute illness [5]. As FDG uptake serves as a correlate for neuronal activity [6], this supports the role of cerebral hypometabolism in the pathogenesis of delirium [5]. 

**Fig. 1:**
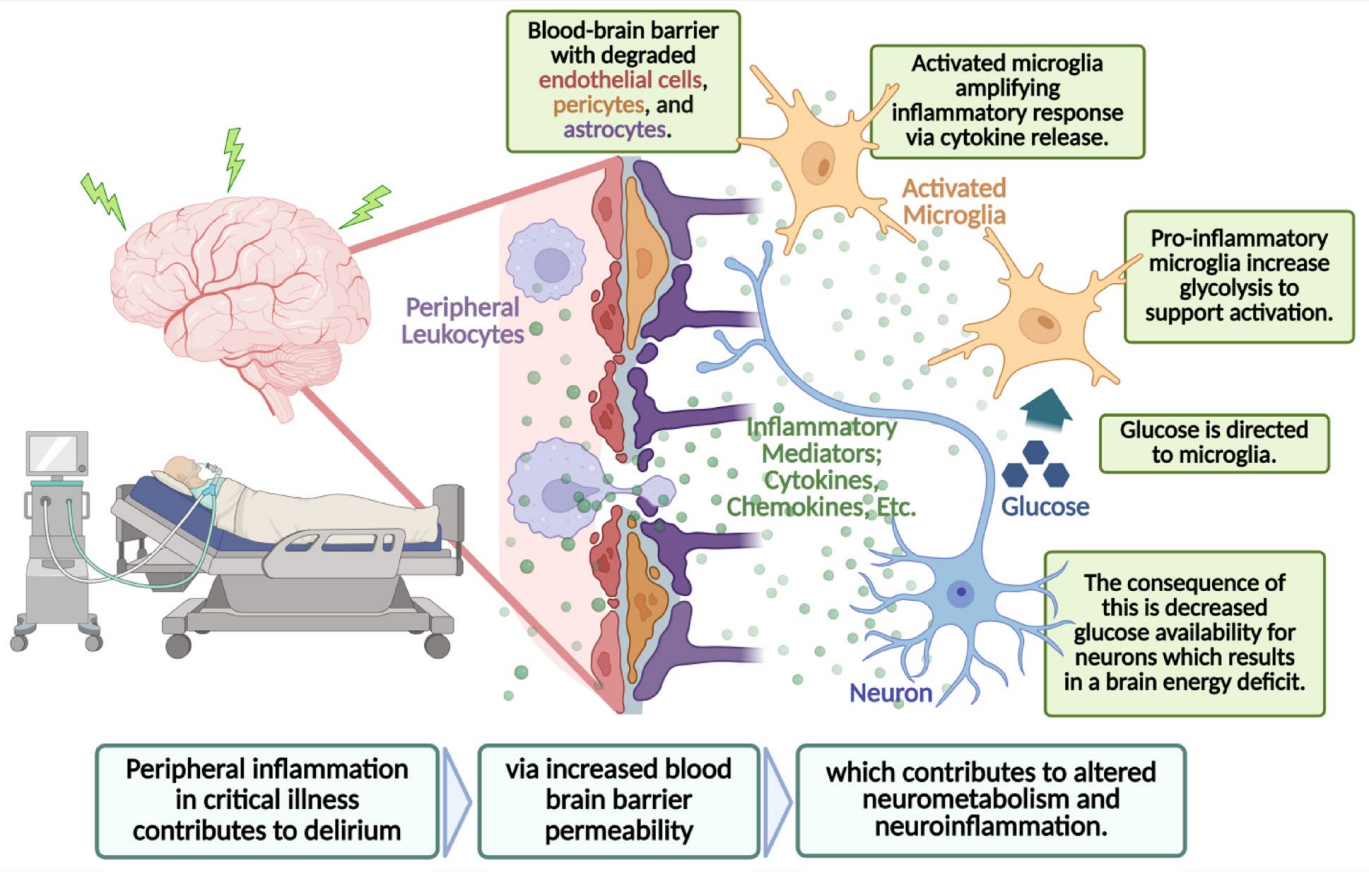
**Conceptual Depiction of the Pathophysiology of Delirium.** Delirium is hypothesized to result from altered glucose metabolism, which occurs as a downstream consequence of peripheral inflammation, leading to a degradation of blood-brain barrier integrity. This results in the activation of resident microglial cells that further amplify the inflammatory response and support their metabolism through exuberant glycolysis, resulting in decreased glucose availability for neurons. Consequently, this creates a cerebral energy deficit, which contributes to delirium. Created with Biorender

Acute brain dysfunction in the ICU, which includes sepsis-associated encephalopathy, postoperative delirium, hypoxia-related encephalopathy, and traumatic brain injury, arises from convergent pathophysiologic mechanisms [7–9]. Delirium is a common clinical manifestation of sepsis-associated encephalopathy; however, sepsis-associated encephalopathy encompasses a broader spectrum of neurologic dysfunction, ranging from delirium to coma [10, 11]. Because delirium is a clinical diagnosis that cannot be made in animals, rodent models can only be said to exhibit delirium-like phenotypes that reflect core domains of delirium, such as inattention, fluctuating arousal, sleep disturbances, EEG disruption, and neuroinflammation [1, 12, 13]. Since much of the most developed mechanistic evidence on the hypothesized pathophysiology of delirium results from murine models of sepsis-associated encephalopathy, and considering that encephalopathy is the brain disorder underlying delirium, we have focused on sepsis-associated encephalopathy in this review as an experimental correlate to delirium [1, 13, 14]. In sepsis-associated encephalopathy, mitochondrial dysfunction occurs in the setting of systemic inflammation, which contributes to increased blood-brain barrier permeability and resultant neuroinflammation [15, 16]. The downstream consequence of this is microglial activation, which amplifies the inflammatory response through the release of pro-inflammatory cytokines [15]. The resultant mitochondrial dysfunction leads to impaired oxidative phosphorylation, decreased adenosine triphosphate (ATP) production, and increased reactive oxygen species production [16, 17]. In response to systemic inflammation, microglia transition to a pro-inflammatory phenotype characterized by increased aerobic glycolysis [15, 18]. This metabolic reprogramming depletes glucose availability for neurons and exacerbates the cerebral energy deficit [18]. Emerging evidence suggests that activated microglia compete with neurons for metabolic substrates during inflammation [19]. Activated microglia exhibit metabolic flexibility, shifting toward increased glycolysis to meet their heightened energy and biosynthetic demands [20]. As a result, this competition for nutrients significantly restricts neuronal glucose uptake, thereby exacerbating the neuronal energy deficit and increasing metabolic stress [21]. We hypothesize that this brain energy deficit contributes to the cognitive and neurological symptoms characteristic of delirium (Fig. 1).

## Ketones as an alternative energy substrate

Relative to its mass, the brain uses more energy than any other organ, consuming 20% of total body energy at rest while comprising just 2% of total body mass [22]. Approximately 75% of energy expenditures support signaling events, representing brain-wide energy use of 30 µmol ATP per gram per minute. In contrast, only 25% of the energy generated is spent on the basic “housekeeping” activities shared by all cells [23]. Under normal physiologic conditions, glucose serves as the primary source of energy for the brain [24]. Unsurprisingly, glucose uptake is a highly coordinated and tightly regulated process. The neurovascular unit, comprised of endothelial cells, pericytes, astrocytes, oligodendrocytes, and microglia, facilitates the delivery of glucose to neurons [18]. 

**Fig. 2:**
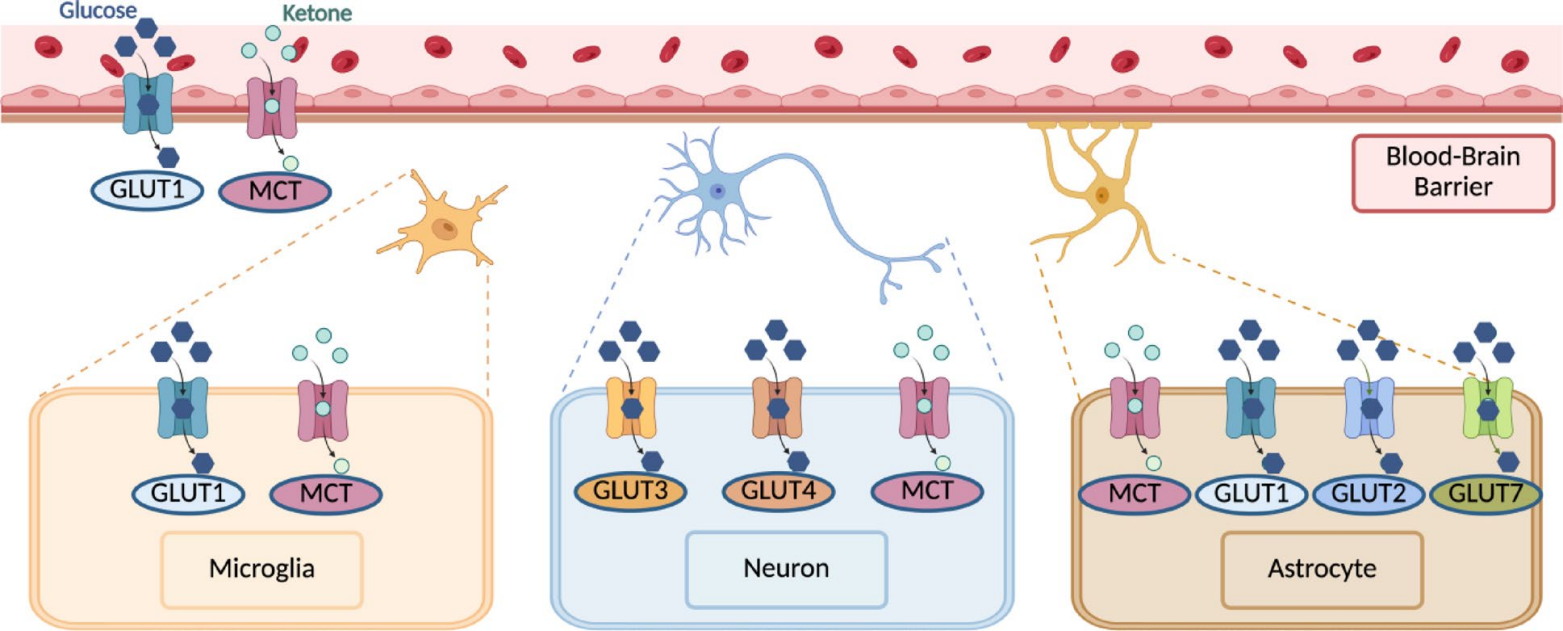
**Distribution of GLUTs and MCTs by Cell Type.** GLUT and MCT are responsible for transporting glucose and ketones from the blood into the brain, respectively. GLUT1 is found on the blood-brain barrier endothelium, microglial cells, and astrocytes. GLUT2 and GLUT7 are found on astrocytes. GLUT3 and GLUT4 are found on neurons. MCTs are found on all cell types. GLUT: glucose transporter; MCT: monocarboxylate acid transporter. Created with Biorender

As cell membranes are impermeable to glucose, glucose transporters (GLUTs) are required to deliver glucose into the cell (Fig. 2) [25]. Different GLUT isoform profiles are found on each component of the neurovascular unit, allowing for tight regulation of glucose uptake [26]. Specifically, GLUT1 is present on the blood-brain barrier endothelium, astrocytes, and oligodendrocytes, GLUT2 and GLUT7 are found on astrocytes, and GLUT3 and GLUT4 are found on neurons [18, 26]. Illustrating the high demand of neurons for glucose, GLUT3 has a higher affinity and higher turnover number relative to other GLUTs. GLUT4 is contained within intracellular vesicles and translocated to the cell membrane during sustained synaptic activity [27]. Neuronal GLUT4 appears to be insulin-responsive and may contribute to cognitive enhancement [28]. Under normal conditions, the aforementioned neurovascular unit and associated GLUTs are able to supply neurons with ample glucose to support energy requirements; however, if glucose demand exceeds delivery, energy reserves are rapidly expended and alternative substrates for ATP generation must be utilized [18]. 

**Fig. 3:**
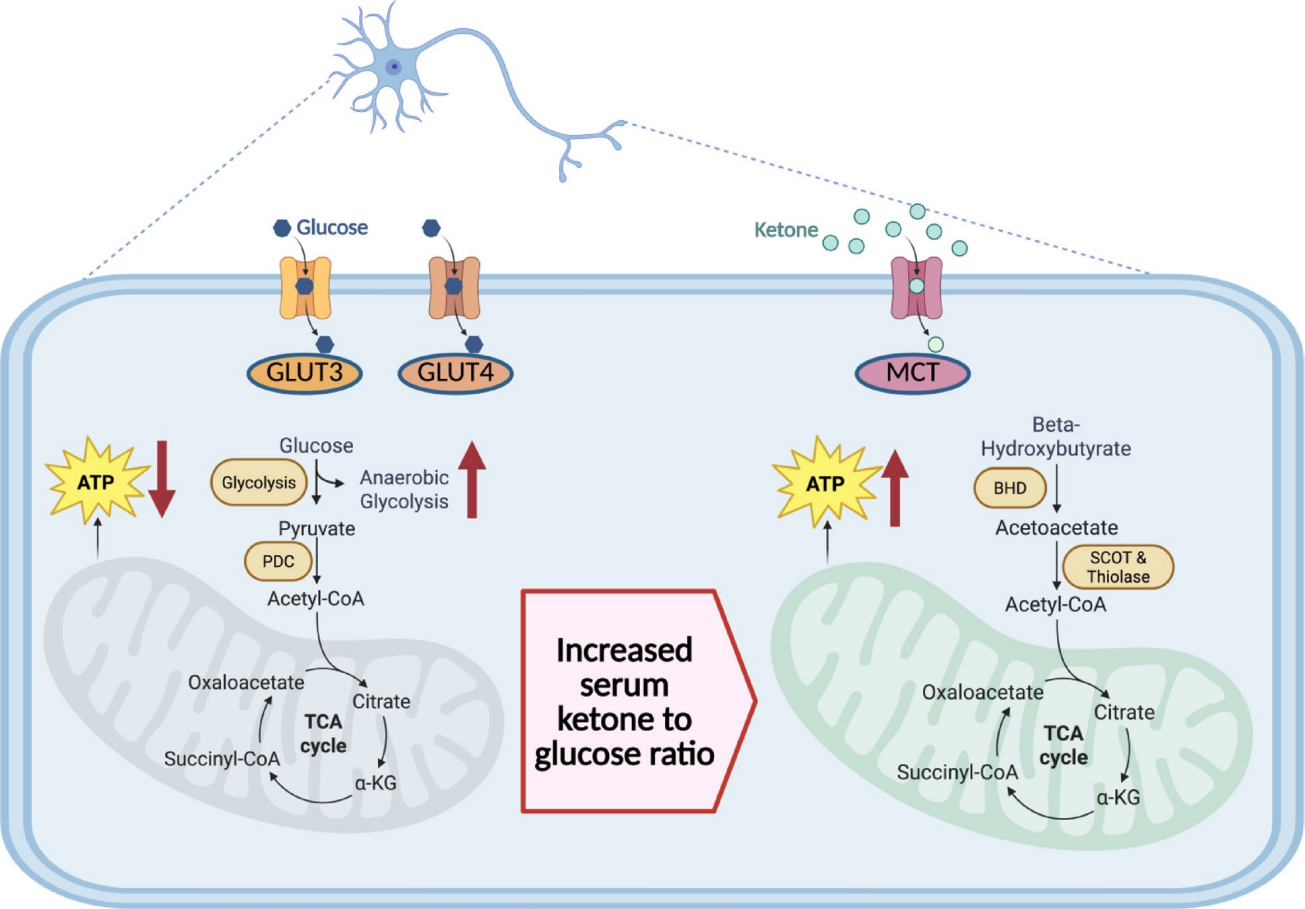
**Ketones are the Preferred Neuronal Energy Substrate when Glucose is Not Available.** When glucose levels are insufficient for CNS metabolism, such as during sepsis, serum ketone concentrations rise and serve as an alternative energy substrate for ATP production in neurons. Ketones enter neurons through MCTs and directly enter the tricarboxylic acid cycle without the energy input that glycolysis requires. Beta-hydroxybutyrate dehydrogenase (BHD) catalyzes the conversion of β-hydroxybutyrate to acetoacetate while reducing NAD + to NADH. Succinyl-CoA:3-oxoacid CoA transferase (SCOT) forms acetoacetyl-CoA, which thiolase cleaves to produce two molecules of acetyl-CoA that then enter the tricarboxylic acid cycle for eventual ATP production. ATP: adenosine triphosphate; GLUT: glucose transporter; MCT: monocarboxylate acid transporter; NAD+: reduced nicotinamide adenine dinucleotide; NADH: oxidized nicotinamide adenine dinucleotide; PDC: pyruvate dehydrogenase complex. Created with Biorender

The ketones acetoacetate and β-hydroxybutyrate are the brain’s secondary source of energy when glucose is not available (Fig. 3) [29]. Under normal conditions, serum ketone concentrations typically range between 100 and 250 µM [30]. Following an overnight fast, ketones provide less than 5% of the brain’s energy; however, concentrations can exceed 5 mM during prolonged fasting, satisfying more than half of brain energy requirements [18]. During periods of fasting, the liver is capable of producing up to 150 g of ketones in a 24-hour period [31]. Like glucose, ketones are dependent on transporters to cross the blood-brain barrier [32]. Unlike GLUTs, which function in response to neuronal activity [33], the monocarboxylate acid transporters (MCTs) that shuttle ketones across the blood-brain barrier and into neurons respond to increases in ketone serum concentration [34]. The rate of transport increases nearly linearly up to a serum ketone concentration of 10 mM, accompanied by a contemporaneous reduction in cerebral glucose utilization, supporting the rapid use of ketones as energy substrates when they are available [35–37]. The brain has limited energy reserves, aside from astrocytic glycogen, so ketone availability for energy production reflects serum levels [36, 38]. In summary, ketones become available as a substrate for ATP production when serum ketone concentrations increase and the ratio of ketones to glucose is elevated [39]. Although a 4-carbon ketone yields approximately 22 ATP, less than the 30–32 ATP produced by a 6-carbon glucose molecule, ketone oxidation is oxygen-efficient and, on a per-carbon basis, provides ATP output comparable to glucose [40, 41]. 

After transport across the blood-brain barrier, acetoacetate and β-hydroxybutyrate are metabolized to acetyl-CoA, thereby directly entering the tricarboxylic acid cycle to produce ATP [18]. In contrast to glycolysis, the conversion of ketones to acetyl-CoA does not require ATP, and ketones only produce ATP via oxidative phosphorylation as they are not metabolized to lactate [18, 42]. In addition to transporting ketones, MCTs facilitate cellular uptake of lactate and pyruvate. Lactate, produced primarily by astrocytes via aerobic glycolysis, serves as a key metabolic substrate for neurons during periods of high synaptic activity or metabolic stress [24, 43]. Elevated CSF lactate observed during delirium may reflect a shift toward glycolytic metabolism in the setting of impaired neuronal oxidative function [44]. In contrast to lactate, ketones bypass glycolysis and enter the tricarboxylic acid cycle directly, offering an alternative and efficient substrate to support neuronal oxidative phosphorylation and restore energy homeostasis [34]. Of note, ketones are not substrates for gluconeogenesis, as they are converted into acetyl-CoA upon uptake, which mammals cannot use to synthesize glucose [30]. Additionally, hepatocytes lack the enzyme required for utilization of ketones for ATP production, Succinyl-CoA:3-oxoacid CoA transferase (SCOT), ensuring that the liver exports the ketones it produces, making them available for energy production in other tissues such as the brain [30, 45]. 

## Neuroprotective effect of ketones

In addition to serving as a substrate for ATP production, ketones support mitochondrial function [46], limit oxidative stress [47], and reduce neuroinflammation [48]. Mitochondrial dysfunction has been implicated in several neurodegenerative diseases, including Alzheimer’s disease, Parkinson’s disease, and Huntington’s disease [49]. Ketones support neuronal health through the activation of pathways involving sirtuins and PGC1α, which induce mitochondrial biogenesis and improve mitochondrial function [46]. Furthermore, ketones support mitochondrial respiration, increase autophagy and cellular repair via stimulation of AMPK, inhibit apoptosis, and limit reactive oxygen species production, thereby contributing to their neuroprotective effect [46, 48, 50]. In addition to reducing reactive oxygen species production, ketones increase antioxidant production by increasing the production of glutathione [51], superoxide dismutase [52], and other endogenous antioxidants [47]. Ketones also provide neuroprotection by attenuating the neuroinflammatory response through the reduction of excessive, maladaptive inflammation [47, 48]. Specifically, β-hydroxybutyrate has been shown to inhibit the NLRP3 inflammasome, resulting in decreased IL-1β and IL-18 production in human monocytes and in murine models [53]. Additionally, it suppresses NF-κB activation subsequent to LPS and TNFα stimulation in murine models [47, 48]. Interestingly, these anti-inflammatory effects were not seen with acetoacetate [47]. Finally, ketones exert neuroprotective effects via inhibiting NMDA receptor stimulation, thereby reducing excitotoxicity caused by excessive glutamate stimulation, as well as increasing the conversion of glutamate to GABA, which contributes to inhibitory-stimulatory neurotransmitter balance [54–56]. In sum, ketones confer a two-fold therapeutic advantage in the setting of nutrient competition. Not only do they support neuronal oxidative phosphorylation by bypassing impaired glycolysis, but they promote anti-inflammatory microglial phenotypes, inhibit inflammasome activation, and support metabolic reprogramming [57]. This dual effect further reduces microglial glucose demand, enhancing neuronal substrate availability.

## Ketones in neurodegenerative disorders

The efficiency of glucose metabolism declines in the aging brain. The association between regional cerebral hypometabolism with mild cognitive impairment and the clinical syndrome of Alzheimer’s dementia (AD) is established [25]. Studies utilizing FDG-PET imaging demonstrate that cerebral hypometabolism correlates with cognitive performance in those at risk of AD and predict the development of mild cognitive impairment, transition from mild cognitive impairment to AD, and development of AD [34]. PET imaging abnormalities can be seen decades before symptom onset [34]. The aging brain may develop a preference for ketones as an energy substrate in response to suboptimal glucose metabolism. In AD, GLUT1 and GLUT3 expression is reduced, decreasing the quantity of glucose transported into the brain at the blood-brain barrier and directly into neurons, respectively [25, 58]. Hypometabolism contributes to neuron death and inflammation with resultant activation of microglia; this further exacerbates inflammation and increases the energy deficit due to the high glucose consumption of microglia [18, 59]. 

In addition to the inadequate availability of glucose for use by neurons, the mitochondrial dysfunction and dysregulated glycolysis that occur in AD inhibit oxidative phosphorylation and result in decreased tricarboxylic acid substrates required for the synthesis of neurotransmitters, such as GABA and acetylcholine, further contributing to the pathology of this disease [58, 60, 61]. The hypometabolism seen in AD appears specific to glucose [25, 39]. Ketones provide less than 5% of brain energy in healthy adults; however, in AD, where cerebral glucose utilization is approximately 25% less than that of healthy matched controls, ketones are being evaluated as an alternative substrate to bridge this energy gap [18, 34, 39, 62]. 

**Fig. 4:**
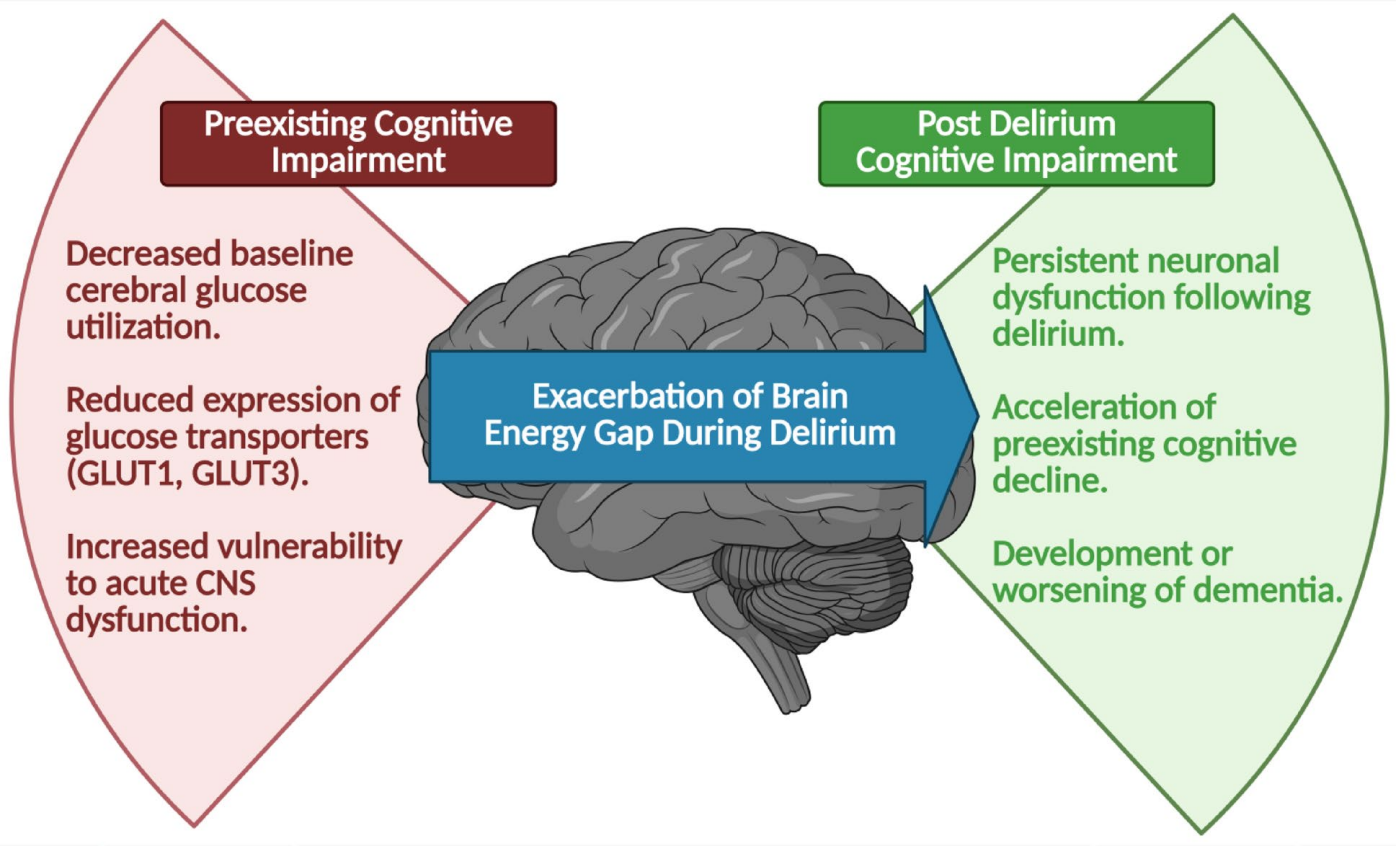
**Brain Energy Gap in Delirium.** Patients with preexisting cognitive impairment, such as mild cognitive impairment or Alzheimer’s dementia, have a baseline brain energy gap due to decreased cerebral glucose metabolism. This energy deficit increases the vulnerability of the aging brain to delirium. Furthermore, the neurometabolic consequences of delirium in those with preexisting cognitive impairment exacerbate the brain energy gap, contributing to the worsening of preexisting cognitive impairment. Created with Biorender

Initial human studies provide support for the use of ketones in AD (Table 1) [25]. In addition to supporting neurometabolism, the beneficial effects of ketone supplementation are hypothesized to arise from reducing neuroinflammation and oxidative stress and via support of mitochondrial function [18]. We hypothesize that the brain energy gap, first described by Cunnane et al., [39] explains the increased susceptibility to developing delirium in the elderly critically ill patient population with preexisting cognitive impairment, and that exacerbation of this brain energy gap as a result of delirium contributes to the progression of preexisting cognitive impairment that is often apparent once delirium resolves (Fig. 4).

**Table 1:** Human studies demonstrating the efficacy of ketogenic interventions

Disease	Intervention	Design	Outcome	Duration	Biomarker	Patients	Reference
MCI	MCTd	RCT parallel	Improved episodic memory, language, executive function	24 weeks	Blood βHB 0.5 mM	83	Fortier M, et al. [63]
MCI	MCTd	RCT parallel	Improved episodic memory, language, executive function, processing speed	24 weeks	Blood βHB 0.5 mM	52	Fortier M, et al. [64]
AD	MCTd vs. placebo	RCT parallel	ADAS-Cog improvement; greater effect in ApoE4–subset	12 weeks	Blood βHB 0.4 mM	152	Henderson ST, et al. [65]
AD	MCTd	Single-arm trial	Improved verbal memory and processing speed	12 weeks	Blood βHB 0.5 mM	20	Ota M, et al. [66]
AD	MCTd vs. placebo	RCT crossover	ADAS-Cog improvement	4 weeks	Blood βHB 0.09 mM	49	Xu Q, et al. [67]
MCI	KD vs. HCLF	RCT parallel	Improved verbal memory	6 weeks	Blood βHB 0.3 mM	23	Krikorian R, et al. [68]
MCI/AD	KD vs. HCLF	RCT parallel	Improved memory composite score	12 weeks	Urine ketones 7.5–10 mg/dL	14	Brandt J, et al. [69]
MCI	KD vs. HCLF	RCT crossover	Improved memory performance	6 weeks	Capillary βHB 0.9 mM	20	Neth BJ, et al. [70]
AD	KD vs. HCLF	RCT crossover	Improved daily function and quality of life	12 weeks	Capillary βHB 0.95 mM	26	Phillips MCL, et al. [71]
AD	KD + MCTd	Single-arm trial	Improved ADAS-Cog	12 weeks	Blood βHB 0.3–0.5 mM	10	Taylor MK, et al. [72]
MCI/AD	Single dose MCTd	RCT crossover	ADAS-Cog improved in ApoE4–subset	1 day	Blood βHB 0.5–0.7 mM	20	Reger MA, et al. [73]
PD + MCI	KD	RCT parallel	Improved lexical access and memory	8 weeks	Blood βHB 0.3 mM	14	Krikorian R, et al. [74]

AD: Alzheimer’s dementia; ADAS-Cog: Alzheimer’s Disease Assessment Scale-Cognitive Subscale; APOE4: Apolipoprotein E4; βHB: β-hydroxybutyrate; HCLF: high-carb low-fat diet; KD: ketogenic diet; MCI: mild cognitive impairment; MCTd: medium-chain triglyceride drink; mM: millimole; PD: Parkinson’s disease; RCT: randomized controlled trial

## Ketones in traumatic brain injury

Ketones have shown promise in traumatic brain injury (TBI), where increased glucose demand and concomitant decreased availability perpetuate secondary neuronal injuries [75]. Ketones are hypothesized to confer neuroprotection by providing an alternative energy source to glucose while restoring normal metabolism [76]. In addition, following TBI, a cascade of impaired oxidative respiration, increased production of reactive oxygen species, and inflammatory changes, similar to what is described above for neurodegenerative disease, results in additional neuronal injury [77]. The NLRP3 inflammasome, an innate immune sensor that responds to damage-associated molecular patterns, contributes to this proinflammatory state, with resultant caspase-1 activation, and cell death [78]. Yet β-hydroxybutyrate suppresses NLRP3 inflammasome activation, and may ameliorate the pro-inflammatory effect [53]. In rats, a ketone drink administered after TBI is neuroprotective; evidenced by reduced neurobehavioral deficits, decreased lesion size, and reduced chronic inflammation measured by GFAP (glial fibrillary acidic protein) staining four weeks after injury [76]. Additional studies have demonstrated the neuroprotective effect of ketogenic diets in rats following TBI, with reductions in cerebral edema and neuron apoptosis, and improved cerebral metabolism [79]. Human trials on the efficacy of ketone administration in TBI are lacking; however, a recent preliminary study demonstrated the safety and feasibility of oral ketone administration in patients with stroke and traumatic brain injury [80]. 

## Acute neurodegeneration in critical illness

The association between ICU delirium and the development of dementia, as well as the acceleration of preexisting cognitive impairment, is well established [1, 81, 82]. Studies have demonstrated that this association is dose-dependent, with longer durations of delirium correlating to worse long-term cognitive outcomes [83–85]. Human biomarker data indicate that direct CNS injury occurs as a consequence of ICU delirium [86], sepsis-associated encephalopathy [87], and postoperative delirium [88]. Specifically, the neurofilament light chain (NfL), a cytoskeletal structural protein of myelinated axons that is detectable in both CSF and serum after neuronal injury, has demonstrated potential in delirium as a prognostic and diagnostic CNS-derived biomarker [86, 88–92]. In a recent study of critically ill patients with respiratory failure, NfL measured at admission was found to be associated with the development of ICU delirium after adjustment for known precipitants of delirium [86]. Elevated NfL at ICU admission has also been associated with the duration of delirium, fewer ICU and hospital free days, and mortality in the critically ill patient population [91]. In sepsis-associated encephalopathy, NfL has demonstrated utility as a prognostic biomarker with elevated levels correlating with the degree of neuronal injury, encephalopathy severity, and worse long-term cognitive outcomes [87, 93–95]. In postoperative delirium, preoperative NfL serum and CSF levels are associated with delirium incidence and severity [88–90, 92]. 

Across multiple clinical contexts, elevated NfL, a marker of acute neuronal injury, is associated with delirium. As delirium duration predicts long-term cognitive decline after critical illness, these findings support a model in which delirium propagates neuronal injury, thereby contributing to post-ICU cognitive impairment. In addition to NfL, GFAP, a cytoskeletal protein found in astrocytes, and brain-derived neurotrophic factor (BDNF), which plays a neuroregulatory role through effects on synaptic plasticity and memory formation, have also been identified as potential CNS-derived serum biomarkers of interest [89, 90, 96, 97]. Studies have demonstrated that GFAP levels rise in conjunction with NfL, potentially improving risk stratification in postoperative delirium [89, 90]. Signals for tau markers and S100B have been demonstrated, but they are less consistent and may be context dependent [87, 98–100]. 

Unlike in neurodegeneration and TBI, trials of exogenous ketone supplementation in ICU delirium are lacking. However, in multiple sclerosis, small trials have demonstrated that a ketogenic diet reduced serum NfL at six months [101], and that NfL reductions at six months were contingent upon achieving a serum β-hydroxybutyrate level of at least 1.0 mM [102]. A study of exogenous ketone ester administration in amyotrophic lateral sclerosis with serum NfL levels at six months as the primary endpoint is currently enrolling patients (NCT04820478) [103]. Since β-hydroxybutyrate has been shown to inhibit the NLRP3 inflammasome [53], and NLRP3 inhibition is associated with lower NfL levels in both human and murine studies [104, 105], NfL serves as a biologically plausible means of measuring ketone-mediated neuroprotection. Considering that elevated CNS-derived biomarkers provide evidence of direct CNS injury in delirium, and that the neuroprotective effects of ketones include reducing neuroinflammation, we hypothesize that exogenous ketone administration will lead to a reduction in the injury signal, as evidenced by lower NfL and GFAP levels in the treatment group.

## Safety and feasibility of ketone supplementation

The neuroprotective effect of ketones was first established over a century ago through the treatment of epilepsy with a ketogenic diet [106]. Historically, experimental and therapeutic states of ketosis were induced by strictly adhering to a high-fat, low-carbohydrate diet or by adding 20 to 30 g of medium-chain triglycerides to a usual diet each day [25]. However, oral ketone esters and ketone salts are now commercially available and safely induce states of ketosis in healthy volunteers [25, 107–109]. Ketone supplementation is increasingly popular in endurance sports due to its ability to serve as an alternative fuel source for oxidative respiration [110]. A study currently recruiting patients evaluating the effect of ketones on brain metabolism and cognitive performance in elderly adults with metabolic syndrome administers a 25-gram ketone monoester drink consumed three times a day for 28 days (NCT04421014); [107] the safety, efficacy, and tolerability of this protocol has been demonstrated [108]. This dose increases serum levels of β-hydroxybutyrate by as much as 4 mM, similar to the level seen after two weeks of starvation, four times greater than what is seen after several weeks of a ketogenic diet, and up to 40 times higher than what is seen following an overnight fast, with levels returning to baseline after approximately 4 h [25]. The level of ketosis reached after an overnight fast via hepatic endogenous ketone production, typically ranging between 100 and 250 µM, is below the level that reliably shifts brain energy utilization in human studies [37, 111]. PET imaging studies show that increased CNS ketone intake rises linearly with serum concentration, with reciprocal reductions in glucose utilization, at approximately 1.0–2.0 mM serum β-hydroxybutyrate [35, 37, 112]. However, as noted in Table 1, longer-term ketogenic interventions appear to confer some neurocognitive benefits at lower concentrations, possibly reflecting the benefits that accrue from cumulative exposure or prolonged maintenance of ketosis [63]. 

Ketone esters are more effective than ketone salts at increasing serum ketone levels without exposing patients to the high sodium content of ketone salts [111]. Nasogastric tube infusions of ketone esters are effective at maintaining β-hydroxybutyrate levels between 2 and 3 mM with infusion rates of 1.1 mM per kilogram per hour following an initial 4.4 mM per kilogram bolus, which results in ketosis equivocal to that seen after administering 4.4 mM per kilogram boluses every three hours [111]. Of note, the pharmacokinetics of various ketone compounds are formulation, dose, and feeding state dependent [110]. After an oral ketone monoester bolus, serum β-hydroxybutyrate levels peak between 30 and 75 min after consumption, and decline over 3 to 4 h, consistent with an effective elimination half-life of 1.5 to 3 h [109, 111]. Despite the established safety of therapeutic ketosis in other settings, the safety of treating critically ill patients, such as those in the intensive care unit requiring invasive support for multi-organ failure, with exogenous ketone ester has not been established. McNelly et al. have demonstrated that the use of a medium-chain triglyceride supplement to induce ketosis was safe in critically ill patients, without exacerbating acidosis or increasing mortality [113]. However, the authors emphasized the need for further studies to establish the safety of exogenous ketone supplementation in this population.

Additional considerations include the bitter taste, mild gastrointestinal symptoms, acid-base effects, salt load, and changes in serum glucose associated with ketone use, which vary across doses and formulations (Table 2). In the critically ill patient population, the bitter taste may be mitigated by delivering ketones via a nasogastric tube or using one of the commercially available products that are made more palatable with flavor additives. Study participants have reported that ketones taken at rest can produce nausea, flatulence, diarrhea, and cramping; however, such symptoms are mild or absent at standard doses [114, 115]. Acid-base effects differ based on the formulation. At standard doses, ketone monoester (R-3-hydroxybutyl R-3-hydroxybutyrate) causes a transient decrease in serum pH and bicarbonate, acid-base shifts have not been reported with butanediol (R-1,3-butanediol), and ketone salts are mildly alkalinizing due to the inorganic cations it is delivered with, representing a salt load between 3 and 6 g [111, 116–118]. However, high doses of butanediol have been shown to cause an anion gap metabolic acidosis in murine models [119]. As β-hydroxybutyrate is a weak organic acid, co-ingestion of sodium bicarbonate in endurance athletes has been demonstrated as an effective means of buffering the transient acid load [111, 120–122]. Finally, exogenous ketones, most notably ketone monoester, have been shown to lower serum glucose [111]. This occurs through complementary mechanisms, including the inhibition of adipose lipolysis, which reduces nonesterified fatty acid substrate availability for hepatic glucose production, and a hepatic redox shift during β-hydroxybutyrate oxidation, where NADH levels increase relative to NAD+, which constrains gluconeogenesis [111, 123–126]. Acute insulin sensitivity changes also contribute; however, they are context dependent and not required for the glucose-lowering effect of ketones [123, 125, 127]. 

In contrast to ketoacidosis, a pathologic state characterized by an anion gap metabolic acidosis with markedly elevated serum ketones, therapeutic ketosis aims for modest increases in β-hydroxybutyrate without the corresponding acid-base and electrolyte disturbances observed in conditions such as diabetic ketoacidosis [111, 128]. The degree of ketosis targeted with exogenous ketone supplementation is similar to that seen in prolonged fasting or strict adherence to a ketogenic diet, states of ketosis without the adverse consequences of acidosis [111, 129, 130]. Like ketones, lactate can serve as a substrate for neuronal energy production, is transported into neurons via MCTs, and allows for metabolic flexibility [43]. Exogenous lactate administration may support cerebral metabolism in TBI [131, 132]. However, as in ketoacidosis, elevated lactate in critical illness is typically indicative of the severity of an underlying pathologic state involving severe acidosis and impaired metabolism, where any neurometabolic benefits that may arise from elevated lactate levels are not realized. Therapeutic ketosis provides an alternative energy source when glucose is unavailable, attenuates inflammation, and reduces oxidative stress, thereby alleviating the brain energy gap without the detrimental effects of pathologic ketoacidosis or lactic acidosis [47]. 

**Table 2:** Ketone formulations and specific considerations

Formulation	Approximate βHB increase per standard dose (mM)	Time to peak concentration (minutes)	Acid-base and electrolyte	Taste and tolerance	ICU specific considerations	References
KME (R-3-hydroxybutyl R-3-hydroxybutyrate)	2–3 (25–27 g dose)	30–75	Acutely lowers blood pH 0.05–0.10; electrolytes unchanged	Bitter; mild GI symptoms possible	Fastest onset, acute decrease in serum glucose	[111, 114, 115, 133]
BDO (R-1,3-butanediol)	1–2 (10–25 g dose)	40–140	Trivial effect on serum acid-base status; electrolytes unchanged	Bitter; mild GI symptoms, but moderate at higher doses	Hepatic metabolism to βHB, exercise caution in liver failure	[111, 116, 118, 122, 134]
β-HB mineral salts (Na/K βHB)	0.5–1 (12–24 g dose)	60–120	Significant salt load, increased urine pH with mild increase in serum pH	GI issues limit higher doses	Consider salt load and fluid balance	[111, 114, 115, 130]

BDO: butanediol; βHB: β-hydroxybutyrate; g: gram; GI: gastrointestinal; ICU: intensive care unit; K: potassium; KME: ketone monoester; Na: sodium

## Proposed clinical trial studying exogenous ketone supplementation in ICU delirium

To evaluate our hypotheses, we propose a prospective, randomized, placebo-controlled pilot study of exogenous ketone ester administration in critically ill patients to assess the safety and efficacy of this novel intervention in reducing the incidence or duration of ICU delirium. Exogenous ketones have been shown to support brain energetics and reduce neuroinflammation [39], directly targeting pathways implicated in the development of delirium. By reducing the duration of delirium or preventing its onset, this research has the potential to improve long-term cognitive outcomes for ICU survivors [83]. We propose enrolling patients aged 50 years or older at the time of ICU admission, with randomization to either an enteral ketone ester treatment group or a calorie-matched glucose-containing placebo for up to 7 days or until ICU discharge, whichever occurs first. The study drug or placebo will be administered first at the time of enrollment, within 12 h of ICU admission, and every six hours thereafter. Ketone administration should be continued after the diagnosis of delirium. In accordance with prior studies, the initial dose of β-hydroxybutyrate will be 25 g; however, subsequent doses will be titrated to maintain serum β-hydroxybutyrate levels between 1.5 and 2.5 mM, with protocolized monitoring of vital signs, serum pH, glucose levels, and adverse gastrointestinal effects [111]. Delirium will be assessed using the CAM-ICU delirium screening tool twice daily [135]. As some patients may screen CAM-ICU positive at enrollment, this proposed study encompasses both prevention and treatment. Subgroup analysis will compare CAM-ICU-positive versus CAM-ICU-negative patients at enrollment to determine the significance of timing for initiating ketone therapy.

This pilot study will assess the safety and tolerability of oral exogenous ketone supplementation in critically ill patients. It will compare the incidence of adverse events, metabolic disturbances, and gastrointestinal intolerance between the ketone and placebo groups. The goal is to demonstrate that ketone administration is well-tolerated, with no significant safety concerns, consistent with prior evidence that oral ketones can be administered safely, even in vulnerable patient populations [80]. Successful completion of this aim will establish a safety profile for ketone use in the ICU, which is essential before adopting this novel therapy for critically ill patients. We hypothesize that patients receiving ketones will have more delirium-free days compared to those receiving a placebo. The primary clinical endpoint will be the number of delirium- or coma-free days. However, we recommend an exploratory analysis of the biological impact of ketone therapy by examining biomarkers associated with delirium and ketone metabolism through serial measurement of serum levels of peripheral inflammatory mediators (including IL-1β, IL-6, IL-8, IL-10, IL-18, CRP, MCP-1, and TNFα) [136–138], metabolic stress assays, β-hydroxybutyrate levels, and markers of CNS injury (including NfL, GFAP, and BDNF). Exclusion criteria should include severe acidosis, fulminant hepatic failure, renal failure requiring dialysis, recent SGLT2 use, diabetic ketoacidosis, refractory shock, pregnancy, uncontrolled ileus, and patients admitted for post-operative monitoring. Future studies can consider the utilization of electroencephalography and fMRI.

## Conclusion

**Fig. 5:**
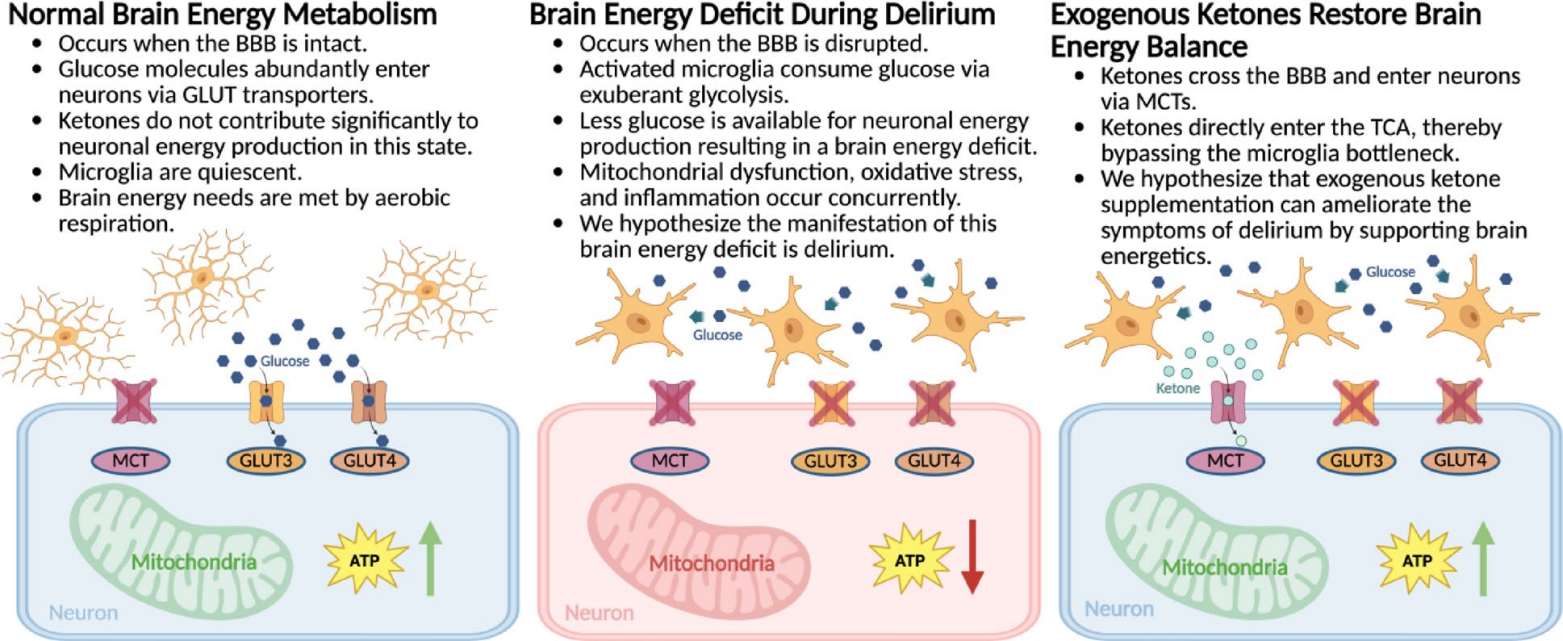
**Overview of the Mechanism by Which Exogenous Ketones Restore Brain Energy Balance in Delirium.** Under normal conditions, glucose efficiently crosses the intact blood-brain barrier through glucose transporters, allowing for sufficient neuronal ATP production via mitochondrial oxidative phosphorylation. During systemic inflammation, blood-brain barrier integrity is compromised, allowing inflammatory cytokines to activate microglia. Once activated, microglia undergo metabolic reprogramming characterized by increased glycolysis, which decreases neuronal glucose availability. Concurrent mitochondrial dysfunction impairs oxidative phosphorylation, resulting in decreased ATP production and increased oxidative stress. We hypothesize that the result of this brain energy deficit is delirium. Exogenous ketones cross the blood-brain barrier and enter neurons via monocarboxylate transporters. Ketones bypass glycolysis and contribute to energy production via oxidative phosphorylation, thereby replenishing neuronal ATP stores, reducing oxidative stress, and alleviating the brain energy deficit. ATP: adenosine triphosphate; GLUT: glucose transporter; MCT: monocarboxylate acid transporter. Created with Biorender

Ketones offer a promising novel therapeutic option for delirium (Fig. 5). By targeting the underlying neurometabolic and neuroinflammatory changes associated with delirium, they support energy production, decrease oxidative stress, and modulate inflammation. Patients with preexisting cognitive impairment, such as those with mild cognitive impairment or Alzheimer’s dementia, exhibit a baseline brain energy gap due to impaired cerebral glucose metabolism. This chronic energy deficit increases the vulnerability of the aging brain to delirium. Furthermore, the neurometabolic consequences of delirium in those with preexisting cognitive impairment exacerbate the brain energy gap, accelerating cognitive decline. The safety, tolerability, and rapid induction of ketosis following oral administration of ketone esters, in addition to the aforementioned theoretical beneficial effects, suggest this may be a therapy that could be initiated upon first signs of ICU delirium or as a potential preventative measure in those patients at risk. Ketones have the potential to transform delirium management and improve patient care; however, randomized clinical trials are required to evaluate the safety and efficacy of oral ketone ester supplementation in reducing the incidence, severity, and duration of delirium in critically ill patients.

## Data Availability

Not applicable.

## Abbreviations

AD: Alzheimer’s dementia
ADAS-Cog: Alzheimer’s disease assessment scale-cognitive Subscale
AMPK: Adenosine monophosphate-activated protein kinase
APOE4: Apolipoprotein E4
ATP: Adenosine triphosphate
βHB: Beta-hydroxybutyrate
BHD: Beta-hydroxybutyrate dehydrogenase
CAM-ICU: Confusion assessment method for the ICU
CSF: Cerebrospinal fluid
GABA: Gamma-aminobutyric acid
GFAP: Glial fibrillary acidic protein
GLUT: Glucose transporter
HCLF: High-carb low-fat diet
IL-18: Interleukin-18
IL-1β: Interleukin-1 beta
KD: Ketogenic diet
LPS: Lipopolysaccharide
MCI: Mild cognitive impairment
MCT: Monocarboxylate acid transporter
MCTd: Medium-chain triglyceride drink
µM: Micromole
mM: Millimole
NAD+: Reduced nicotinamide adenine dinucleotide
NADH: Oxidized nicotinamide adenine dinucleotide
NF-κB: Nuclear factor kappa-light-chain-enhancer of activated B cells
NfL: Neurofilament light chain
NLRP3: Nod-like receptor family pyrin domain containing 3
NMDA: N-methyl-D-aspartic acid
PD: Parkinson’s disease
PDC: Pyruvate dehydrogenase complex
PGC1α: Peroxisome proliferator-activated receptor gamma coactivator 1-alpha
RCT: Randomized controlled trial
SCOT: Succinyl-CoA:3-oxoacid CoA transferase
TBI: Traumatic brain injury
TNFα: Tumor necrosis factor alpha
